# The Role of Sleep in Cardiovascular Disease

**DOI:** 10.1007/s11883-024-01207-5

**Published:** 2024-05-25

**Authors:** Vita N. Jaspan, Garred S. Greenberg, Siddhant Parihar, Christine M. Park, Virend K. Somers, Michael D. Shapiro, Carl J. Lavie, Salim S. Virani, Leandro Slipczuk

**Affiliations:** 1 Division of Cardiology, Montefiore Health System/Albert Einstein College of Medicine, Bronx, NY USA; 2 Department of Cardiovascular Medicine, Mayo Clinic, Rochester, MN USA; 3 Center for Preventive Cardiology, Section On Cardiovascular Medicine, Wake Forest University Baptist Medical Center, Winston-Salem, NC USA; 4 Ochsner Clinical School, John Ochsner Heart and Vascular Institute, The University of Queensland School of Medicine, New Orleans, LA USA; 5 Office of the Vice Provost (Research), The Aga Khan University, Karachi, Pakistan; 6 Division of Cardiology, The Texas Heart Institute/Baylor College of Medicine, Houston, TX USA; 7 Michael E. DeBakey Veterans Affairs Medical Center and Baylor College of Medicine, Houston, TX USA

**Keywords:** Sleep, Cardiovascular disease, Prevention, Obstructive sleep apnea

## Abstract

**Purpose of Review:**

Sleep is an important component of cardiovascular (CV) health. This review summarizes the complex relationship between sleep and CV disease (CVD). Additionally, we describe the data supporting the treatment of sleep disturbances in preventing and treating CVD.

**Recent Findings:**

Recent guidelines recommend screening for obstructive sleep apnea in patients with atrial fibrillation. New data continues to demonstrate the importance of sleep quality and duration for CV health.

**Summary:**

There is a complex bidirectional relationship between sleep health and CVD. Sleep disturbances have systemic effects that contribute to the development of CVD, including hypertension, coronary artery disease, heart failure, and arrhythmias. Additionally, CVD contributes to the development of sleep disturbances. However, more data are needed to support the role of screening for and treatment of sleep disorders for the prevention of CVD.

## Introduction

Sleep is increasingly recognized as a key component of cardiovascular (CV) health. Humans spend approximately 30% of their lives sleeping [1]. Additionally, CV disease (CVD) is the leading cause of morbidity and mortality in the United States [2]; therefore, it is critical to understand the relationship between sleep and CV health and disease.

In 2022, the American Heart Association (AHA) expanded their “Life’s Simple 7,” which constitute important determinants of cardiovascular health, to “Life’s Essential 8,” by adding sleep as one of the eight core components that define optimal CV health [3••]. Healthy sleep was added to the list of well recognized components of good CV health, including: diet, exercise, avoidance of nicotine, maintenance of a healthy weight, healthy blood lipid levels, healthy blood glucose levels, and normal blood pressure, upon a foundation of psychological health and social determinants of health.

Epidemiological studies have demonstrated the important role of sleep duration in CV health [4]. Ultimately, the AHA decided to include sleep duration in their “Life’s Essential 8” due to the influence of sleep on each of the other seven components of CV health.

While the AHA specifically focuses on sleep duration, there is overwhelming evidence that sleep quality and the presence of primary sleep disorders are also important mediators of CV health. A prospective study of the MESA (Multi-Ethnic Study of Atherosclerosis) cohort revealed that CV health scores that incorporated aspects of sleep health, including sleep duration, daytime sleepiness, and obstructive sleep apnea (OSA) better predicted CV disease risk than those that merely incorporated the original “Life’s Simple 7” [5•].

**Fig. 1:**
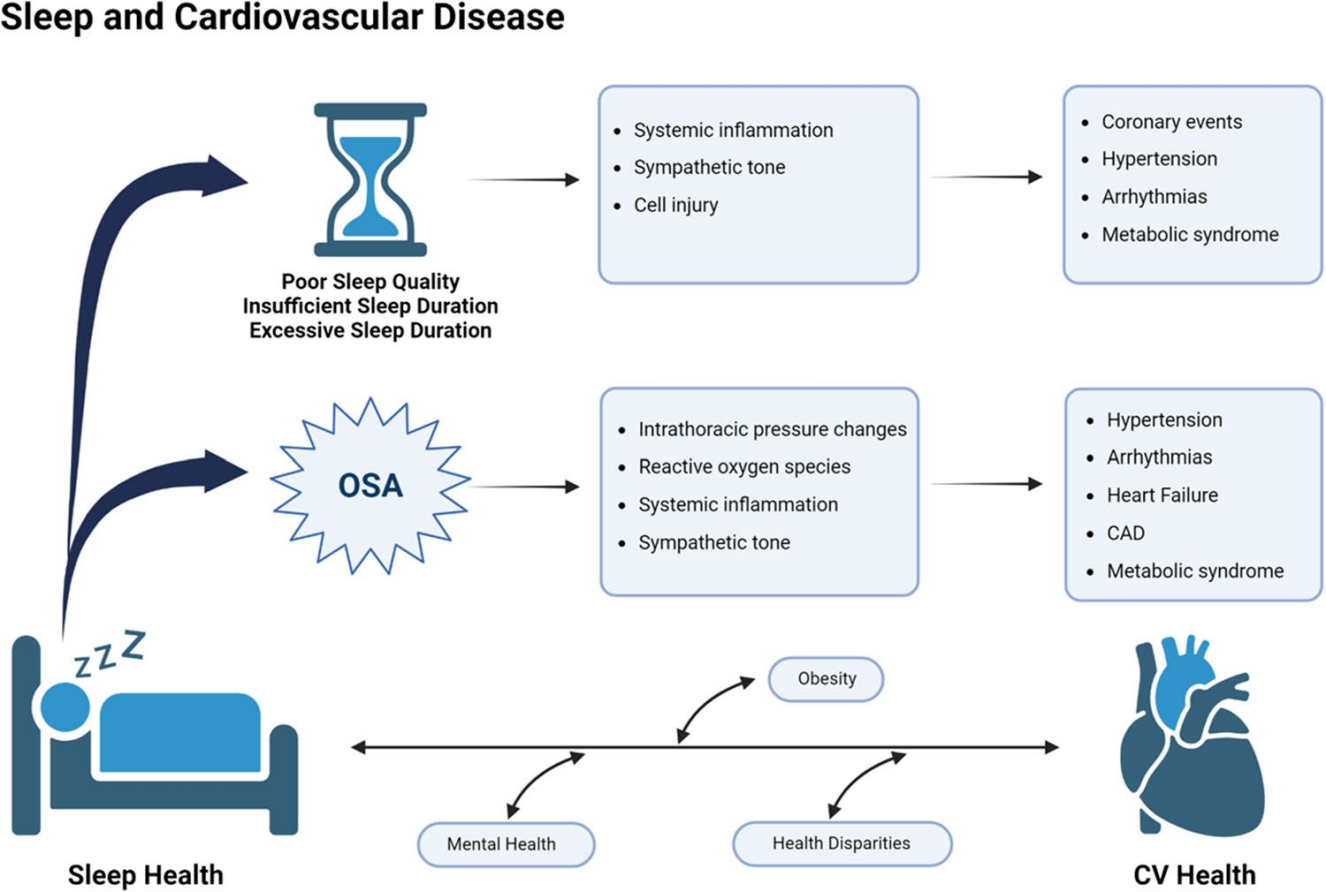
Central Illustration- Overview of the links between sleep health and cardiovascular health. *OSA* obstructive sleep apnea, *CAD* coronary artery disease, *CV* cardiovascular. Created with BioRender.com. Central illustration demonstrating the links between sleep health and cardiovascular health. OSA = obstructive sleep apnea, CAD = coronary artery disease, CV = cardiovascular

In this review of the literature, we summarize the data demonstrating how perturbations of normal sleep are associated with increased risk of CVD. Additionally, we demonstrate the links between OSA and CVD. Finally, we illustrate the bidirectional relationship between sleep quality and CVD (Fig. 1).

## Sleep Quality and Duration as a Risk Factor for CVD

### Pathophysiology

Proper sleep, defined as 4–5 sleep cycles of light, deep, and rapid eye movement (REM) sleep, is essential to maintaining cardiometabolic homeostasis [6]. Disruptions in both sleep duration and quality have been implicated as risk factors for CVD [7–9]. This may be due to immune dysregulation, increased sympathetic tone, chronic endocrine stress response, and endothelial dysfunction [10].

The hypothalamic–pituitary–adrenal (HPA) axis, which is tightly linked to circadian rhythms, is a principal mediator of the neuroendocrine stress system and likely plays a key role in the propagation of cardiometabolic diseases [10]. Research has demonstrated that after just a few nights of sleeping only 3–4 h, subjects experienced a significant hormonal imbalance, with morning cortisol levels decreasing by approximately 30% and afternoon levels increasing by around 40% [11, 12]. This observation was noted in those undergoing acute and chronic sleep restriction, defined as 3 or 4 h in bed, as well as sleep fragmentation, defined as being woken up multiple times overnight [13–15]. Ultimately, this stress response leads to increased heart rate, decreased heart rate variability, increased blood pressure, and increased secretion of catecholamines, all of which are risk factors for or associated with coronary artery disease (CAD) [16–18].

Several analyses demonstrated an association between sleep restriction and both increased heart rate and decreased heart rate variability, suggesting a decrease in cardiac parasympathetic and/or increase in sympathetic tone [19–23]. One cross-sectional study of 30 young males during university final exams demonstrated that sleep deprivation, defined as sleep duration less than 80% of baseline over 4 weeks, was associated with increased plasma norepinephrine levels (315 to 410 pg/ml, p < 0.05) [24]. Autonomic dysregulation leads to a perpetuation of sleep issues like insomnia and fragmented sleep, as well as obesity, insulin resistance, and ultimately, increased risk for CAD [10, 25].

Chronic inflammation is likely a mediating factor in the connection between sleep quality and the development of CAD. Inflammation is a key factor in the development of CAD [26]. The physiologic circadian rhythm directly regulates immune cells and inflammatory cytokines, including tumor necrosis factor-α (TNF-α), and interleukins (IL): IL-1, IL-2, IL-6, and IL-10. Several of these inflammatory markers have been associated with sleep duration and have thus been implicated in CAD mediated by poor sleep [27, 28]. Studies on the impact of sleep duration and TNF-α have shown that sleep restriction generally increases TNF-α levels [29–31]. The Cleveland Family Study, a population level evaluation, showed that each hour less of sleep on polysomnography was associated with an 8% increase in TNF-α. However, other studies have shown that sleep deprivation did not consistently increase TNF-α levels [32, 33]. Sleep deprivation studies have also linked restricted sleep with increased inflammation through increased IL-6 levels [34–36].

High-sensitivity C-reactive protein (hs-CRP), an acute phase reactant that plays a critical role in in opsonizing low-density lipoprotein cholesterol by macrophages in atherosclerotic plaque, has been linked with sleep duration [28, 37]. Epidemiological studies suggest that hs-CRP is a predictor of CVD events [38, 39]. Several studies have demonstrated an association between decreased sleep and increased hs-CRP [40, 41]. Additionally, large epidemiological studies including The Nurses’ Health Study, The Cleveland Family Study, Whitehall Study, and Study of Women’s Health Across the Nation, revealed significant associations between increased sleep duration and elevated hs-CRP levels, especially in women. This association persisted even after adjusting for demographic, socioeconomic, and health risk factors [35, 42–44]. A meta-analysis of 72 studies, showed that sleep disturbances and longer sleep duration are associated with higher levels of hs-CRP (ES 0.12: 95% CI 0.05 – 0.19; and ES 0.17: 95% CI 0.01 – 0.34, respectively) and IL-6 (ES 0.20: 95% CI 0.08 – 0.31; and ES 0.11: 95% CI 0.02 – 0.20, respectively). However, short sleep duration was not associated with increased inflammatory markers [27].

Elevated fibrinogen levels have also been linked with CVD. Among 3,471 participants in the PESA (Progression of Early Subclinical Atherosclerosis) cohort study, lower fibrinogen levels were associated with regression of subclinical atherosclerosis [45]. Multiple large cohort studies, including one analysis of 3,942 post-menopausal women as part of the Women’s Health Initiative, revealed an association between prolonged sleep and elevated fibrinogen levels [36, 46]. This study also implicated fibrinogen as a mediating factor between prolonged sleep duration and CVD.

Lastly, endothelial dysfunction is an independent predictor of CVD risk [10, 47]. Randomized studies have shown significant impairment in both arterial and venous endothelial function after several days of sleep restriction [48]. Total sleep deficit also hindered arterial endothelial and microvascular function in healthy subjects [49, 50].

### Sleep Duration and CV Health

Insomnia and sleep restriction are linked to poor CVD outcomes [51–57]. A prospective Dutch cohort study of 20,432 men without CAD who slept less than or equal to 6 h per night and had poor sleep quality had a 79% higher risk of CAD (HR: 1.79 [1.24–2.58]) after adjusting for risk factors compared to those with > 7 h of sleep per night (Table 1) [58]. Similarly, an analysis of a Chinese cohort of 60,586 subjects showed that both short sleep duration and poor sleep quality were associated with an increased risk of CAD (HR 1.13, 95% CI: 1.04–1.23; and HR: 1.40, 95% CI: 1.25–1.56, respectively) [59].

**Table 1:** Studies of Sleep and Cardiovascular Disease

Study	Design	Number of Patients	Inclusion	Exclusion Criteria	Primary Outcome	Results
Hoevenaar-Blom 2011 (MORGEN) [58]	Prospective cohort study	20,432	Residents of 3 towns in the Netherlands aged 20–65 years	History of CVD, Pregnant women, those with insufficient data or follow up	Coronary events	Short sleepers had increased coronary events compared to normal sleepers
Lao 2018 [59]	Prospective cohort study	60,586	Taiwanese adults aged 40 or older	Known history of CVD or cerebrovascular disease, diabetes, thyroid disease, pulmonary disease, hepatitis, CKD, cancer, cirrhosis	Incident coronary heart disease	Short and poor quality sleep are associated with increased risk of CAD
Strand 2016 [61]	Prospective cohort study	392,164	Taiwanese adults aged 20 or older who attended a health checkup program from 1994–2011	Insufficient data on sleep quality/duration, confounding variables	Death from cardiovascular disease	Both short and long sleep durations were associated with increased risk of cardiovascular mortality
Makarem 2022 (MESA) [5•]	Prospective cohort study	1920	Adults	Clinical CVD	CVD incidence	CVH scores including sleep better predict CVD risk than those that do not
Peppard 2000 (Wisconsin Sleep Cohort) [82]	Prospective cohort study	709	Employees of Wisconsin state agencies	Pregnancy, decompensated cardiopulmonary disease, airway cancer, recent surgery of the upper respiratory tract, unusable sleep study data, prior stroke, treatment for sleep-disordered breathing	Hypertension incidence	Sleep disordered breathing is associated with development of hypertension
Macek 2023 [101]	Cross sectional study	124	Age > 18 years, indication for coronary CT	Known ischemia, stroke, renal failure, thyroid disorders, active malignancy, active inflammation	CAC score	Severity of OSA correlated with CAC score
McEvoy 2016 (SAVE) [105]	Randomized controlled trial, parallel-group, open-label with blinded end-point assessment	2717	Adults age 45–75 with moderate to severe OSA and coronary or cerebrovascular disease	Planned revascularization procedure, NYHA III-IV HF, prior CPAP use, Cheyne-Stokes respiration	Composite of death from cardiovascular causes, myocardial infarction, stroke, unstable angina, HF, or transient ischemic attack	CPAP did not prevent cardiovascular events in patients with moderate to severe OSA and established CVD
Peker 2016 (RICCADSA) [106]	Prospective randomized controlled trial, open-label, blinded evaluation	244	Newly revascularized CAD and AHI < 5 or > 15/h	Existing OSA, AHI 5.0–14.9 per hour, predominantly central apneas with Cheyne-Stokes respiration	First event of repeat revascularization, myocardial infarction, stroke, or cardiovascular mortality	CPAP did not significantly reduce long-term adverse cardiovascular outcomes
Peker 2020 (RICCADSA) [107]	Prospective randomized controlled trial, open-label, blinded evaluation	353	Patients who presented with ACS after revascularization and AHI < 5 or > 15/h	Existing OSA, AHI 5.0–14.9 per hour, predominantly central apneas with Cheyne-Stokes respiration	First event of repeat revascularization, myocardial infarction, stroke, or cardiovascular mortality	Significant risk reduction in those who used CPAP for > 4 h/day
Kaneko 2003 [115]	Randomized controlled trial	24	Reduced LVEF on optimal medical therapy, OSA	Primary valvular disease, pacemaker, unstable angina, myocardial infarction, recent cardiac surgery	LVEF and systolic blood pressure	Treatment of coexisting OSA reduces systolic blood pressure and improves LV systolic function
Bradley 2005 (CANPAP) [117]	Randomized controlled trial, open-label	258	Adults with HF on optimized medical therapy with central sleep apnea	Pregnancy, myocardial infarction, unstable angina, or cardiac surgery within 3 months, OSA	Combined rate of death from all causes	CPAP improved LVEF and increased 6 min walk distance; did not affect survival
Cowie 2015 (SERVE-HF) [118]	Randomized, parallel-group, event-driven	1325	Adults with LVEF < 45%, AHI > 15, one HF hospitalization within 24 months, central sleep apnea	COPD, CPAP use, life expectancy < 1 year unrelated HF, recent TIA or stroke, significant valvular disease, cardiac surgery, PCI, myocardial infarction, or unstable angina within 6 months, pregnancy	First event of death from any cause, lifesaving cardiovascular intervention, or unplanned hospitalization for worsening HF	Adaptive servo-ventilation did not significantly affect the primary end point, though did increase all-cause and cardiovascular mortality

*AHI* apnea–hypopnea index, *LVEF* left ventricular ejection fraction, *OSA* obstructive sleep apnea, *CPAP* continuous positive airway pressure, *CAC* coronary artery calcium, *CVD* cardiovascular disease, *CAD* coronary artery disease, *COPD* chronic obstructive pulmonary disease, *HF* heart failure, *TIA* transient ischemic attack

While decreased sleep is associated with CVD, accumulating evidence suggests that increased sleep duration is also linked to poor CV health. A meta-analysis of 15 studies demonstrated that both shorter sleep duration (usually defined as ≤ 6 h per night) and longer sleep duration (usually > 8 h per night) were associated with significantly increased risk of CAD and stroke [60•]. Subsequently, a large cohort study of 392,164 adults followed for 18 years found that those who slept less than 4 h/night and greater than 8 h/night had a 34% and 35% increased risk of dying from CAD, respectively, when compared with those that slept 6–8 h/night. A statistically significant U-shaped association between sleep duration and CVD mortality was only observed in female subjects and those aged 65 years and above [61]. A meta-analysis of 15 studies showed that both short and long sleep duration were associated with increased CVD mortality (RR 1.25, 95% CI 1.06–1.47 and 1.26 95% CI 1.11–1.42, respectively) [4]. Moreover, when stratified by sex, the negative effects of sleep duration on CVD mortality were only observed in women. Consistent with these findings, others have noted that the extremes of sleep duration increase the risk of CV death in patients with prior myocardial infarctions (MI) and are associated with prevalence of subclinical atherosclerosis as evidenced by coronary artery calcium scores (CAC) [8, 62, 63].

While the U-shaped relationship between sleep duration and CVD is mirrored by similar trends in inflammatory markers, the underlying mechanisms are not completely understood. Possible rationales include the effects of confounding factors such as depressive symptoms, socio-economic status, unemployment, and limited physical activity associated with longer sleep durations [64, 65].

### Disparities in Sleep Health

Many environmental factors impact sleep health, including exposure to stressors, tobacco, alcohol, pollutants, and allergens [66]. Therefore, certain communities may be more prone to poor sleep than others. Several studies have investigated racial and ethnic differences in sleep health. For example, an analysis of data from the National Health Interview Survey of 155,203 participants revealed that compared to White participants, Filipino individuals were less likely to get adequate sleep (> 7 h) [67]. Additionally, a retrospective analysis of a large United States cohort revealed that relative to White adults, Black adults were more likely to have short sleep duration, and that there were significant interactions with income, sex, and geographic location [68]. In addition to racial and ethnic disparities in sleep health, there are sex disparities in sleep. A meta-analysis of 31 studies including 1,265,015 participants revealed that women were more likely than men to experience insomnia [69]. Additionally, a randomized controlled crossover study of 4 h versus 8 to 9 h of sleep, short sleep was associated with increases in both daytime and nighttime BP, predominantly in women [70]. More studies are needed to determine how these differences in sleep health translate to disparities in CV health. This is especially important as sleep health seems to be deteriorating on a population level [71].

### Confounding and Mediating Factors

While sleep health has been linked with cardiovascular health, there are several factors that may confound or mediate this relationship. Sleep disturbances frequently occur in conjunction with numerous psychiatric conditions, including major depressive disorder and acute stress disorder [72]. There is a bidirectional relationship between sleep health and mental health [73]. Thus, mental health may act as an important mediating factor or confounding variable when analyzing the relationship between sleep health and CV health. Additionally, there are complex multidirectional relationships between obesity, mental health, sleep health, and CV health [74–76], which could further confound or mediate the relationship between sleep health and CV health. Therefore, it is difficult to determine how much of the link between sleep and CV health is primarily due to the effects of sleep quality and duration versus due to the complex interplay among many interrelated factors.

### Sleep Quality and the Prevention of CVD

While there is a plethora of evidence that poor sleep health is associated with CVD, there are significantly less data supporting the role of addressing sleep health for the primary prevention of CVD. A prospective analysis of the MESA Sleep Study revealed that participants with an increased CV health score, which included increased multidimensional sleep health, had lower incident CVD risk [5•]. Additionally, a recent study of 6,251 participants concluded that low delta wave entropy, a marker of poor sleep quality, was associated with increased risk of CVD and CVD mortality [77]. This suggests that there may be a role for addressing sleep health for the primary prevention of CVD. Ultimately, the AHA determined that despite the paucity of evidence directly indicating that improved sleep duration reduces CVD incidence, there is enough evidence supporting the links between sleep duration and cardiometabolic health and health outcomes to include sleep duration in the formal definition of CV health [3••]. Notably, the AHA did not directly include sleep quality as part of this definition, though this may change in the future as more data becomes available.

## OSA as a Risk Factor for CVD

### Acute Physiological Effects of OSA

Obstructive sleep apnea (OSA) is characterized by repetitive upper airway closure during sleep, resulting in cycles of apnea and hypopnea associated with oxygen desaturations [78••]. These repetitive cycles of apnea and hypopnea have many direct physiologic consequences. For example, the intermittent hypoxia and reoxygenation results in oxidative stress through the production of reactive oxygen species, resulting in systemic inflammation and endothelial dysfunction [79]. Several inflammatory markers, including cytokine IL-6 and hs-CRP have been found to be elevated in patients with OSA compared with obese controls, with improvement after treatment with continuous positive airway pressure [79, 80]. Recurrent arousals, along with intermittent hypoxia, are thought to result in increased sympathetic activation [79]. Additionally, inspiration against a closed upper airway results in large intrathoracic pressure swings, which contributes directly to shear stress on the aorta and other intrathoracic vessels [79]. Ultimately, intermittent hypoxia, intrathoracic pressure changes, and sympathetic activation have many implications for CVD, including links to hypertension, arrhythmias, heart failure (HF), and CAD.

### OSA as a Risk Factor for Hypertension

Hypertension and OSA frequently co-occur in the same patients. More than 30% of patients with hypertension have concomitant OSA [81]. A prospective study of the Wisconsin Sleep Cohort of 709 participants revealed a dose–response association between apnea–hypopnea index (AHI) and the presence of hypertension [82]. There is a particularly strong association between resistant hypertension, defined as suboptimal blood pressure control despite the use of at least three antihypertensives including a diuretic, and OSA. A recent meta-analysis of 7 studies including 2,541 patients demonstrated that patients with OSA were at more than three times increased risk of resistant hypertension (OR 3.34 [2.44, 4.58]) even when adjusting for associated risk factors, including obesity, age, and smoking status [83•].

Unfortunately, despite strong evidence that OSA is associated with hypertension, the impact of OSA treatment on blood pressure (BP) has been relatively modest. A randomized controlled trial (RCT) of patients with OSA without daytime sleepiness randomized to CPAP or no CPAP demonstrated no difference in incidence of hypertension or CVD [84]. Several studies have demonstrated a reduction in systolic BP of 3–5 mm Hg [85, 86]. Interestingly, one meta-analysis revealed that reduction in BP was only seen in studies that had > 3 month follow-up, suggesting that perhaps the benefits of continuous positive airway pressure (CPAP) are more chronic and require longer follow-up time to appreciate improvements in hypertension [85]. Finally, the CRESCENT (Cardiosleep Research Program on Obstructive Sleep Apnoea, Blood Pressure Control and Maladaptive Myocardial Remodeling—Non-inferiority Trial) study, a recent RCT of patients with moderate to severe OSA and hypertension found that mandibular advancement devices were non-inferior to CPAP in reduction in BP, with a reduction in mean arterial pressure of 2.5 mmHg in 6 months [87]. As of 2021, the AHA recommends screening for OSA in patients with resistant or poorly controlled hypertension [78••]. Screening can be completed quickly, easily, and reliable with the STOP-BANG questionnaire [88].

### OSA as a Risk Factor for Arrhythmias

OSA contributes to rhythm disturbances at the level of the sinus node, atria, and ventricles [89]. Atrial fibrillation (AF) is the most common arrhythmia associated with OSA, with a prevalence of approximately 35% [90•]. Animal models suggest that this is likely a result of atrial oxidative stress [91]. Additionally, increased vagal tone during apneic events results in a shortened effective refractory period, which promotes atrial fibrillation in a porcine model [91]. A meta-analysis of 16 studies demonstrated increased likelihood of developing AF with increased AHI [90•]. A separate meta-analysis of nine studies including 14,812 patients concluded that CPAP reduced the risk of AF recurrence or progression by 63% in patients with OSA compared to patients with OSA not on CPAP [92]. Screening for OSA is recommended in patients with recurrent AF after cardioversion or ablation [78••], though two RCTs concluded that there was no evidence that CPAP treatment of OSA after cardioversion [93] or ablation [94] resulted in reduced AF recurrence. The 2023 American College of Cardiology/AHA/American College of Chest Physicians/Heart Rhythm Society Guidelines for the Diagnosis and Management of AF provide a grade 2b recommendation of screening for OSA in patients with AF, though they note that the role of treatment of OSA to maintain sinus rhythm is uncertain [95••].

In addition to atrial arrhythmias, patients with OSA are prone to sick sinus syndrome, sino-atrial block, and tachycardia-bradycardia syndrome [96]. Among patients with OSA, bradycardia was present in 25% during the daytime and 70% during the night [97]. This has significant clinical implications, as the European Multicenter Polysomnographic Study showed an excessively high prevalence of undiagnosed OSA (59%) in patients who required pacing [98]. There are insufficient data to assess whether treatment of the underlying OSA would have obviated the need for pacing in these patients.

Finally, patients with OSA are predisposed to ventricular arrhythmias. This is thought to be related to the imbalance of sympathetic and parasympathetic tone [96]. Patients with OSA are more likely to experience sudden cardiac death overnight, which is a stark contrast from the general population, which has a nadir from midnight to 6 a.m. [99], suggesting a role of OSA in the development of ventricular arrhythmias.

### OSA and CAD

OSA is thought to be a risk factor for the development of CAD due to oxidative stress and systemic inflammation. Interestingly, OSA may also have protective effects against the development of CAD as cycles of hypoxia could promote the generation of increased coronary collateral blood flow. A recent study of the UK Biobank suggests a gene-environment interaction mediating the risk of CAD in patients with OSA [100]. This study suggested involvement of various pathways including vascular endothelial growth factor and TNF in the gene-by-environment interaction in the development of CAD in patients with OSA.

One study of 124 participants undergoing coronary artery computed tomography angiography for clinical indications revealed that OSA with an AHI > 14.9 was a predictor of a high CAC score (> 400 Agatston Units) with a sensitivity of 62% and specificity of 80% [101]. Prior observational studies have shown increased CAD events in patients with OSA [102–104].

There is controversy whether treatment of OSA reduces the risk of CAD. The Sleep Apnea Cardiovascular Endpoints (SAVE) trial, a RCT of 2,717 patients with moderate-to-severe OSA with CAD or cerebrovascular disease with a mean follow up of 3.7 years, demonstrated no benefit of CPAP in reducing CVD events [105]. Additionally, a separate RCT of patients with OSA and newly revascularized CAD showed no significant difference in rates of repeat revascularization, MI, stroke, or CVD mortality in those who did versus did not receive treatment with CPAP [106]. Further analysis of the same study population found that those with CPAP use for > 4 h per day had significant risk reduction in repeat revascularization, MI, stroke, or cardiovascular mortality during a median 4.7-year follow up (HR 0.17, 95% CI 0.03–0.81; p = 0.03) [107]. Ultimately, more data is needed to better understand the importance of CPAP on the development and progression of CAD in patients with OSA.

### OSA and HF

OSA is quite common among HF patients, with 48% of HF with reduced ejection fraction (HFrEF) and 36% of HF with preserved ejection fraction (HFpEF) patients having an AHI of at least 15 per hour in a German registry [108]. In this registry, OSA comprises 69% of these cases in HFrEF patients, and 81% in HFpEF patients, with central sleep apnea (CSA) comprising the remaining cases.

There are several mechanisms by which OSA causes adverse hemodynamic consequences for HF patients. An occluded airway reduces intrathoracic pressure with inspiration, increasing venous return and right ventricular distension, while reducing left ventricular (LV) filling, increasing LV transmural pressure, and increasing afterload [109, 110]. Afterload and myocardial oxygen demand also increase due to the sympathetic stimulus and hypertension induced by recurrent hypoxia, which can result in LV remodeling and hypertrophy over time [111, 112]. There is evidence of a bidirectional relationship, as fluid accumulation in the neck is thought to be a contributor to the development of OSA in HF patients [113].

OSA has been shown to be a risk factor for mortality in patients with HF, and the mortality rate for patients with HF and sleep-disordered breathing (SDB) in the United States has been rising over the last decade [114]. A small RCT of 24 patients with OSA and an ejection fraction (EF) less than 45% tested the addition of CPAP to optimal medical therapy, and after one month, showed a significant improvement in mean systolic BP (-10 mmHg, p = 0.02), reduction in LV end-systolic diameter (-2.8 mm, p = 0.009), and recovery of LVEF (+ 8.8%, p < 0.001) as assessed by echocardiography [115]. While there are small studies testing intermediate outcomes, there are no RCTs to date assessing CPAP therapy in HF patients with OSA [116].

Three major RCTs tested positive airway pressure for the treatment of CSA in HF patients, and neither showed a mortality benefit. The Canadian CPAP for Patients with CSA and HF (CANPAP) trial, which randomized 258 patients with both CSA and HFrEF on optimal medical therapy for the time period, with an average EF of 24.5%, to CPAP and no CPAP [117]. While there were small but statistically significant increases in EF and the six-minute walk test, there were no differences in hospitalizations, quality of life, death, or heart transplantation, and the trial was stopped prematurely. The Treatment of Predominant CSA by Adaptive Servo Ventilation in Patients With Heart Failure (SERVE-HF) trial was an RCT that randomized 1325 patients with an LVEF of 45% or less to adaptive servo-ventilation, a non-invasive ventilatory therapy that delivers dynamically adjusted air pressure, compared to medical therapy alone [118]. The composite primary endpoint of all-cause mortality, lifesaving CV intervention, or unplanned HF hospitalization was not significant; however, adaptive servo-ventilation (ASV) was associated with a significant increase in all-cause and CVD mortality. Finally, the ASV for SDB in Patients with HFrEF (ADVENT-HF) trial, an RCT that randomized patients with HFrEF and SDB to ASV versus standard care demonstrated that while ASV was safe and effective for treatment of SDB, it did not result in a reduction in all cause mortality or a composite of CV outcomes [119].

### OSA and Metabolic Syndrome

OSA has long been investigated as a potential independent contributor to the CVD risk associated with the metabolic syndrome [120]. Patients with OSA have significantly higher BP, serum glucose, triglycerides, cholesterol, and low-density lipoprotein cholesterol [121]. Sleep-disordered breathing was independently associated with glucose intolerance, insulin resistance, and diabetes in population based studies [122–124]. Additionally, treatment of OSA is associated with improvement in cardiometabolic and inflammatory parameters, including reduced BP, total cholesterol, apolipoprotein B, insulin resistance index, malondialdehyde, and TNF-α [125]. Animal models and clinical studies provide evidence that OSA contributes to the metabolic syndrome via metabolic, sympathetic, and inflammatory pathways [126].

### Impact of Treatment of OSA on CVD Outcomes

There are multiple device, lifestyle, and procedural interventions that have been shown to successfully treat OSA, but there is limited evidence to support an improvement in CVD outcomes [78••, 127]. CPAP is the mainstay of therapy for OSA, and it is associated with a large improvement in the AHI, sleepiness, quality of life, and cognitive measures, and it is associated with a small reduction in systolic blood pressure [128–130]. As discussed above, the CANPAP and SAVE trials did not demonstrate a reduction in cardiovascular events or mortality with CPAP. Mandibular advancement devices are oral appliances that can reduce OSA symptom severity, reduce systolic BP, and improve quality of life, but they are not as efficacious at reducing the AHI compared to CPAP [95••, 131].

Guidelines support weight loss to a body mass index (BMI) less than 25 in obese patients, in addition to other lifestyle interventions including exercise, and positional therapy [132]. The Sleep Action for Health in Diabetes (AHEAD) compared an intensive lifestyle intervention to routine education in obese diabetics with OSA, which resulted in a 10.2 kg weight loss (P < 0.001) and an improvement in the AHI by 9.7 events per hour (P < 0.001) [133]. While very few of these patients were receiving CPAP therapy, the positive effect of weight loss on OSA severity among patients on CPAP was shown in a subsequent RCT [134].

Pharmacologic or surgically supported weight loss can also improve outcomes in OSA. The Satiety and Clinical Adiposity Liraglutide Evidence (SCALE) Sleep Apnea RCT tested liraglutide 3.0 in a randomized, double-blind trial of non-diabetics and showed a statistically significant improvement in weight and AHI [135]. Another RCT compared traditional weight loss to bariatric surgery in 60 obese patients with OSA, and despite a weight loss of 27.8 kg in the surgery group (compared to 5.1 kg with lifestyle intervention, P < 0.001), the improvement in the AHI was not statistically significant [136]. This suggests that the relationship between OSA severity and obesity is non-linear, and that there are other factors at play, such as the anatomy of the upper airway. However, as obesity is associated with poor cardiovascular health, weight loss is likely helpful for both OSA and CVD outcomes [137].

The main surgical procedures used in management of OSA include uvulopalatopharyngoplasty and other soft tissue reduction procedures, maxillomandibular advancement, and hypoglossal nerve stimulation [127]. However, these are invasive procedures and there is limited evidence that they improve CVD outcomes.

## CVD as a Risk Factor for Poor Sleep

Finally, while poor sleep is associated with CVD, CVD is also associated with poor sleep quality. Patients with HF are prone to the development of CSA due to the effect of pulmonary venous congestion on vagal irritation receptors, resulting in reflex hyperventilation and dysregulation in the ventilatory control system due to high hypercapnic responsiveness [138–140]. This then leads to oscillating breathing patterns with periods of central apnea and/or hypopnea followed by periods of hyperventilation. This waxing-waning breathing pattern is commonly referred to as “Cheyne-Stokes respiration” (CSR) [141, 142]. Prior studies have reported a prevalence of 33–40% among patients with HF [143, 144]. CSA and CSR cause disrupted sleep with frequent arousals and overall reduced time spent in REM and slow wave sleep [142]. This manifests as symptoms of daytime sleepiness, paroxysmal nocturnal dyspnea, and fatigue [141]. HF patients with CSA have higher mortality and morbidity compared to those without CSA. CSA was found to be an independent risk factor for overall mortality, with studies showing the cumulative survival and transplant free progression was significantly lower in HF patients with CSA compared to HF patients without CSA [145, 146]. There was also a higher predisposition for fatal arrhythmias, possibly via sympathetic nerve activation that can be exacerbated by the frequent arousals during the periodic breathing patterns in CSA [141, 142].

Additionally, CVD is associated with poor sleep health indirectly through impacts on mental health. Depression, which is significantly more common in patients with CVD, is associated with poor sleep. The relationship between depression and CVD is complex and bidirectional, with biological, environmental, and behavioral links [147].

## Conclusion

Sleep is increasingly recognized as an important component of CV health. There is a complex bidirectional relationship between sleep and CVD. Perturbations to normal sleep as well as primary sleep disorders have systemic effects, including changes in autonomic tone and inflammation, which contribute to the development of a wide range of CV disorders, including hypertension, rhythm disturbances, metabolic syndrome, and coronary artery disease. There is also an interplay with sleep quality and mental health, which has implications for cardiovascular disease. Finally, CV diseases can also impact sleep quality, both directly through the development of CSA, and indirectly mediated by effects on mental health. Recent guidelines are beginning to incorporate screening and treatment of sleep disorders for the treatment of cardiovascular disease. More data is necessary to determine the role of screening and addressing sleep disturbances for the prevention of cardiovascular disease.

## Data Availability

No datasets were generated or analysed during the current study.
